# Dendritic Cells Are the Major Antigen Presenting Cells in Inflammatory Lesions of Murine Mycoplasma Respiratory Disease

**DOI:** 10.1371/journal.pone.0055984

**Published:** 2013-02-04

**Authors:** Xiangle Sun, Harlan P. Jones, Nicole Dobbs, Sheetal Bodhankar, Jerry W. Simecka

**Affiliations:** Department of Molecular Biology and Immunology, University of North Texas Health Science Center in Fort Worth, Fort Worth, Texas, United States of America; University of Texas at Tyler, United States of America

## Abstract

Mycoplasmas cause chronic respiratory diseases in animals and humans, and to date, development of vaccines have been problematic. Using a murine model of mycoplasma pneumonia, lymphocyte responses, specifically T cells, were shown to confer protection as well as promote immunopathology in mycoplasma disease. Because T cells play such a critical role, it is important to define the role of antigen presenting cells (APC) as these cells may influence either exacerbation of mycoplasma disease pathogenesis or enhancement of protective immunity. The roles of APC, such as dendritic cells and/or macrophages, and their ability to modulate adaptive immunity in mycoplasma disease are currently unknown. Therefore, the purpose of this study was to identify individual pulmonary APC populations that may contribute to the activation of T cell responses during mycoplasma disease pathogenesis. The present study indeed demonstrates increasing numbers of CD11c^−^ F4/80^+^ cells, which contain macrophages, and more mature/activated CD11c^+^ F4/80^−^ cells, containing DC, in the lungs after infection. CD11c^−^ F4/80^+^ macrophage-enriched cells and CD11c^+^ F4/80^−^ dendritic cell-enriched populations showed different patterns of cytokine mRNA expression, supporting the idea that these cells have different impacts on immunity in response to infection. In fact, DC containing CD11c^+^ F4/80^−^ cell populations from the lungs of infected mice were most capable of stimulating mycoplasma-specific CD4^+^ Th cell responses *in vitro*. *In vivo*, these CD11c^+^F4/80^−^ cells were co-localized with CD4^+^ Th cells in inflammatory infiltrates in the lungs of mycoplasma-infected mice. Thus, CD11c^+^F4/80^−^ dendritic cells appear to be the major APC population responsible for pulmonary T cell stimulation in mycoplasma-infected mice, and these dendritic cells likely contribute to responses impacting disease pathogenesis.

## Introduction

Mycoplasma infection is a leading cause of pneumonia worldwide. In the United States, alone, *Mycoplasma pneumoniae* accounts for 30% of all cases of pneumonia [1]–[3]. Mycoplasma disease is also associated with the exacerbation of other respiratory diseases, such as asthma [4]. *Mycoplasma pulmonis* causes a naturally occurring murine chronic respiratory disease with high morbidity and low mortality. *M. pulmonis* is an excellent animal model of *M. pneumoniae,* allowing for the characterization of immune responses during the pathogenesis of mycoplasma respiratory disease. Both *M. pulmonis* and *M. pneumoniae* respiratory infections cause rhinitis, otitis media, laryngotracheitis, and bronchopneumonia. In terms of histopathology, both diseases are characterized by the accumulation of mononuclear cells along the respiratory airway [2], [5]–[8]. This suggests that the activation and recruitment of immune cells are important in the development of both acute and chronic states of the disease.

It is clear that part of the adaptive immune system contributes to the pathology, while part is protective against *M. pulmonis* infections. Studies using immunodeficient mice demonstrated that lymphoid responses can be immunopathologic, contributing to the severity of pulmonary disease [9]–[11]. Furthermore, pulmonary T cell responses are central to the outcome of disease [12], [13]. The development of chronic inflammatory lesions in lungs do not develop until between 10 to 14 days after infection, corresponding with increases in T cell numbers and their activation. The depletion of T helper cells (Th) results in less severe lung disease, demonstrating that a Th cell response contributes to disease pathology in the lung [14]. Further studies indicate that Th2 responses are responsible for the immunopathology in mycoplasma disease [15], [16]. However, adaptive immunity can still prevent dissemination of infection and can promote resistance to infection and disease [10]. In addition, Th1 cell responses appear to promote resistance to infection and dampen inflammatory responses [15]. CD8^+^ T cells and CD25^+^ T_reg_ cells can also reduce the severity of inflammatory disease [14] (A. Odeh and J.W. Simecka, unpublished data). Thus, pulmonary T cell activation and the mechanisms that regulate these responses are instrumental in the pathogenesis of mycoplasma respiratory disease of the lower respiratory tract.

Because of their central role in development of T cell responses, antigen-presenting cells (APC) should be influential in determining immune-mediated pathology or protection from mycoplasma induced chronic respiratory disease. There is little to no information on the role of APC populations, particularly dendritic cells (DC), during generation of immune and inflammatory responses in any mycoplasma respiratory disease. Both DC and pulmonary macrophages may be involved in the generation of harmful and/or beneficial pulmonary immune responses [17]–[19]. Of interest, DC are extremely potent antigen-presenting cells, which can activate both Th and cytotoxic T cells, and are found in lungs [20]–[26], as well as other tissues. They are capable of modulating the type of T cell responses generated [27]. However, studies suggest that the resident DC in lungs are immature [28] and are not as effective in antigen presentation. This indicates that the naïve lung is typically not a site where immune responses are initiated. Nevertheless, numbers of DC in lungs can increase in inflammatory disease [29]–[31], and studies suggest that DC are critical in the generation of allergic and asthmatic responses [32]–[35] and therefore may play a role in inducing immune-mediated inflammatory disease. Presumably, pulmonary DC during respiratory diseases are capable of driving T cell responses within the lung that are contributing to the pathogenesis of these inflammatory reactions. Thus, we hypothesized that pulmonary DC are likely to play a pivotal role in the activation and retention of effector T cells associated with the inflammatory lesions of mycoplasma pneumonia.

The purpose of this study was to determine the potential of cell populations in the lung to perpetuate T cell responses in the chronic inflammatory lesions characteristic murine mycoplasma pneumonia. Clinical disease, chronic inflammatory lesions, and increases in pulmonary T cells do not develop until 7 days after *M. pulmonis* infection (usually between 10 to 14 days after infection) [14], [36], and therefore, the focus was on the changes in DC and macrophage populations in lungs of mice which were uninfected with those infected with mycoplasma 14 days previously. For these studies, we took advantage of cell surface markers to fractionate the different antigen presenting cell populations. In particular, F4/80 is found primarily on macrophages, but can also be expressed on a small fraction of DC [37]. CD11c is also often used to isolate and characterize DC; however, other cells, such as alveolar macrophages can express both F4/80 and CD11c molecules [38]. The present study shows that CD11c^+^ F4/80^−^ cells, most likely DC, were the major APC population responsible for T cell stimulation in the lungs of mycoplasma-infected mice, and these *in vivo* interactions likely contribute to the immune responses that impact disease pathogenesis. These results further our understanding of the immune mechanisms involved in disease pathogenesis, which will likely lead to more effective vaccines against mycoplasma respiratory diseases.

## Results

### There is an increase in activated DC and macrophage populations in the lungs of mycoplasma-infected mice

There is an increase in T cells in lungs of infected mice [14], and we hypothesized that there may be a concurrent increase in antigen presenting cells, e.g. DC and macrophages. To examine whether changes in numbers of DC or macrophages occur during disease pathogenesis, mice were infected with *M. pulmonis* and compared to the control mice inoculated with sterile broth medium. At 14 days after infection, mononuclear cells were collected from the lungs, and the cells were stained with fluorescently labeled anti-CD11c and/or -F4/80 antibody (Ab) for flow cytometry analysis. The total number CD11c^+^ F4/80^−^ cells (CD11c^+^, DC-enriched), CD11c^−^ F4/80^+^ cells (F4/80^+^, macrophage-enriched) and CD11c^+^ F4/80^+^ cells [double positive, alveolar macrophage-enriched [38]] were determined.

**Figure 1:**
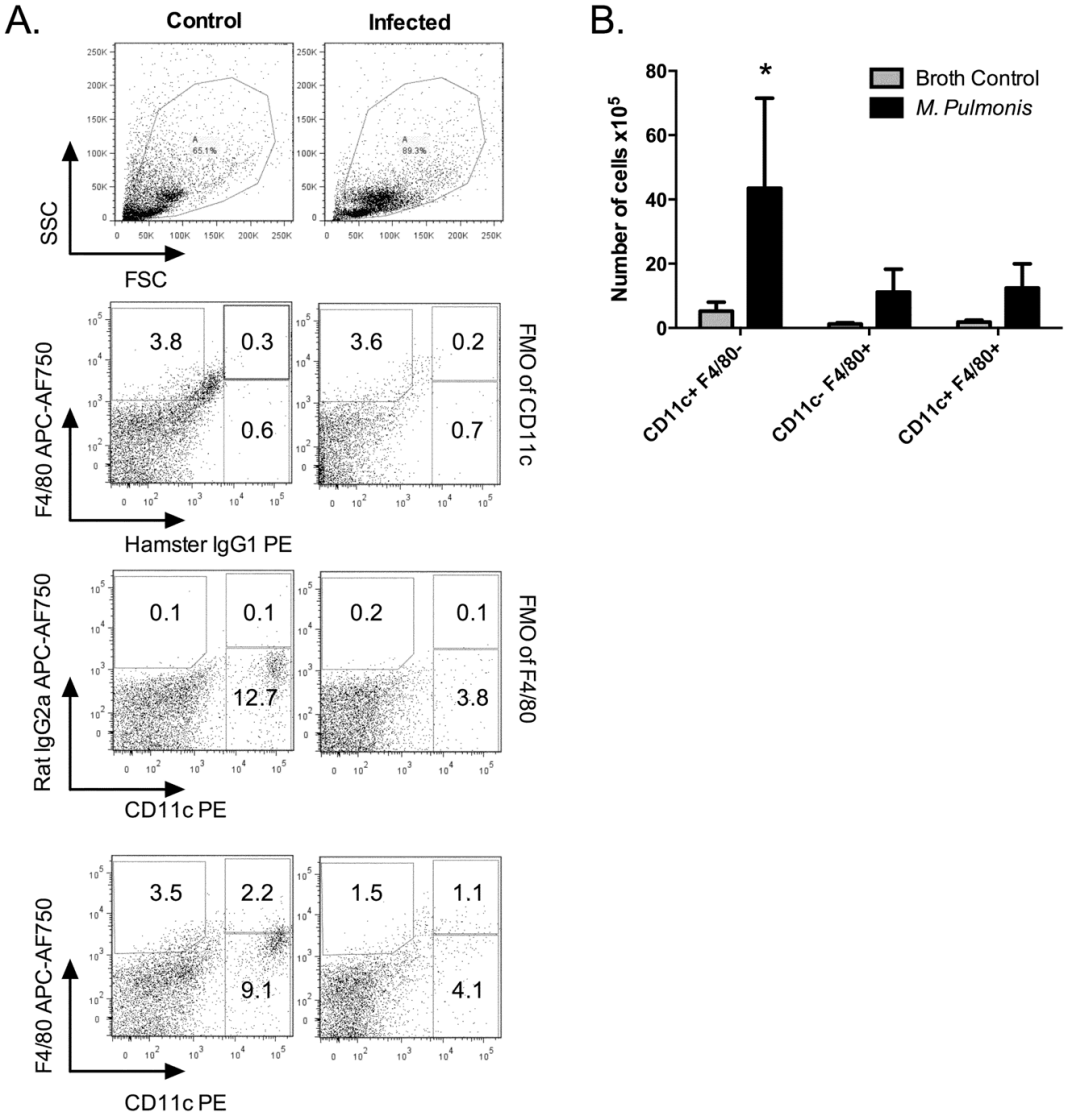
The numbers of CD11c^+^ and F4/80^+^ cells increased in lungs of mycoplasma-infected mice. Mice were infected with *M. pulmonis* or broth as control. At day 14 after infection, lung mononuclear cells were isolated from individual mice and analyzed by flow cytometry following staining with fluorescent antibodies. (A) Dot plots showing the gating scheme used to determine the frequency of CD11c^+^ F4/80^−^, F4/80^+^ CD11c^−^ and CD11c^+^ F4/80^+^ cells. The criterions of CD11c positive populations were determined by FMO plus isotype control of CD11c; for this, the cells were stained with all the antibodies but anti-CD11c was replaced by isotype control antibody. The same strategy was applied to determine F4/80 positive population. The numbers in the dot plots indicate the mean percent of the total population within the specified gate. (B) The numbers of CD11c^+^F4/80^−^ cells, CD11c^−^ F4/80^+^ cells and CD11c^+^ F4/80^+^ cells were determined based on the percentage of these three cell populations and the total numbers of cells isolated from the lungs of these mice. An asterisk (*) indicates a significant difference (*P*≤0.05, n = 9 from two separate experiments) between *M. pulmonis* infected and broth inoculated (Control) mice.

At 14 days after infection, there was a significant increase in each of the DC and macrophage populations recovered from the lungs of infected mice (Fig. 1). The large influx of other mononuclear cells in response to mycoplasma infection resulted in an apparent lower percentages of DC and macrophages populations. However, there was still about a 4-fold increase in the numbers of CD11c^+^ F4/80^−^ cells in lungs at 14 days after infection. There were also similar increases in the numbers of CD11c^−^ F4/80^+^ cells and double positive (CD11c^+^ F4/80^+^) cells. There was not a change in cell numbers in lungs at 7 days after infection (data not shown). In CD11c^+^ F4/80^−^ cells (Table 1), the percentage of cells expressing MHC II along with CD11b (phenotypically similar to conventional dendritic cells, cDC [39]) increased by 14 days after infection. Less than 10% of the cDC (CD11c^+^ F4/80^−^ MHC II^+^ CD11b^+^ cells) population from lungs of control or infected mice also expressed CD8 (data not shown). In contrast, there was a relative decrease in B220^+^CD11b^−^cells in the CD11c^+^F4/80^−^ population (phenotypically similar to plasmacytoid dendritic cells, pDC [40], [41]). Thus, CD11c^+^ cell (DC-enriched, in particular the cDC population), F4/80^+^ (macrophage-enriched), and double positive (alveolar macrophage-enriched) cell populations contributed to the cellular response in the lungs during mycoplasma disease pathogenesis.

**Table 1:** Subpopulations of CD11c^+^F4/80^−^ cells from lungs of control and infected mice.

Populations of CD11c^+^F4/80^−^ cells	Control	Infected
CD11b^+^MHC II^+a^	15.4% (1.1) b	37.8% (4.1)*
CD11b^+^MHC II^int^	6.7% (0.25)	23.9% (3.6)*
CD11b^+^MHC II^hi^	8.6% (0.95)	13.9% (1.1)*
B220^+^	14.5% (1.1)	7.5% (1.7)*
CD11b^−^ B220^−^	42.0% (1.1)	22.7 (3.2)*

a CD11b^+^MHC II^+^ cells were further differentiated by their expression of either high (^hi^) or intermediate (^int^) levels of MHC II.

b Percentage (%) of CD11c^+^ F4/80^−^ cells that express the respective phenotype (SEM) (n = 9, two experiments).

* Denotes P≤0.05 from % of cells from lungs of control mice.

As the lungs were previously shown to have few mature resident DC [28], changes in numbers of mature DC would support their role in disease. The co-stimulatory molecules, CD80, CD86, CD40 and MHC II, are required for antigen presentation by macrophages and DCs [42], [43], and the expression of these molecules increase as these cell populations mature and become more capable of antigen presentation [44], [45]. Therefore to examine the maturation changes in the APC populations, we compared the expression of MHC II, CD40, CD80 and CD86 proteins on the surface of cell populations from lungs of control (uninfected, sterile broth inoculated) and mycoplasma-infected (14 days after infection) mice using immunofluorescent staining and flow cytometry.

**Figure 2:**
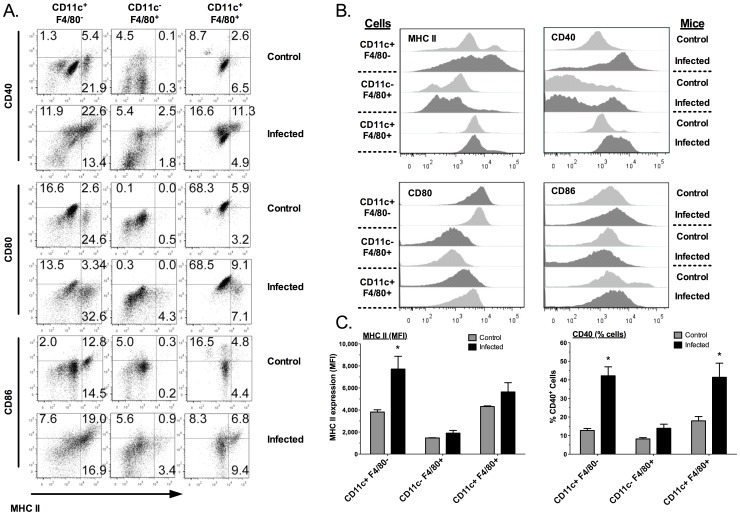
Increased expression of CD40 and MHCII on pulmonary CD11c^+^ cells after mycoplasma infection. Pulmonary mononuclear cells from broth control mice or 14 days after *M. pulmonis* infected mice were analyzed by flow cytometry as indicated in Fig 1A. (A) Dot plots show MHCII versus CD40, CD80 or CD86 expression after gating on CD11c^+^F4/80^−^, CD11c^−^ F4/80^+^ or CD11c^+^ F4/80^+^ cell populations. The numbers in the dot plots indicate the mean percent of the total population within the specified gate. (B) The MHCII, CD40, CD80 and CD86 expression level in pulmonary CD11c^+^F4/80^−^, CD11c^−^ F4/80^+^ or CD11c^+^ F4/80^+^ cells from broth inoculated (Control) or *M. pulmonis* infected mice are shown in histograms. (C) MHCII^+^ expression (mean fluorescence index, MFI) on the APC populations were compared between cells recovered from the lungs of uninfected and *M. pulmonis* infected mice. A similar comparison of the percentages of CD40^+^ cells in the three cell populations is shown. An asterisk (*) indicates a significant difference (*P*≤0.05, n = 9 from two separate experiments) between *M. pulmonis* infected and broth inoculated (Control) mice.

There were significant changes in the pulmonary APC populations as a result of mycoplasma disease (Fig. 2). CD11c^+^ F4/80^−^ cells (DC) from the lungs of infected mice had significantly higher MHC II expression and percentage of cells expressing CD40, as compared to cells recovered from control mice. In the other two APC populations, there was no change in MHC II expression after infection, but there was a significant increase in the percentage of CD40 expressing double positive (CD11c^+^F4/80^+^) cells found in infected lungs. Furthermore, CD11c^+^F4/80^−^ cells had the highest percentage of MHC II^+^ CD40^+^ cells after infection (about 23%), whereas about 12% of CD11c^+^F4/80^+^ cells expressed both molecules. The macrophage-enriched population, which expressed both markers, was negligible (less than 3%). Although CD80 and CD86 were expressed on each of the cell populations, there was little increase in the expression of CD80 or CD86 in any of the cell populations in response to infection. However, CD11c^+^F4/80^−^ cells had the highest percentages of cells that expressed both MHC II and CD86/CD80 molecules. Thus, MHCII, CD80 and CD86 mainly reside on pulmonary CD11c^+^ cells and upregulated in response to *M. pulmonis* infection compared to F4/80^+^ cells, which is consistent with an increased potential to promote T cell activation by CD11c^+^ cells.

### CD11c^+^ F4/80^−^ DC and CD11c^−^ F4/80^+^ macrophages are localized within Th cell containing inflammatory infiltrates in lungs of infected mice

In the previous study, there was a dramatic increase in the numbers of macrophages and DC in the lungs of infected mice, and these results corresponded with our earlier study [14] demonstrating an increase in pulmonary Th cells resulting from infection. Using immunofluorescence and confocal microscopy, the possible co-localization of CD11c^+^ F4/80^−^ DC and CD11c^−^ F4/80^+^ macrophages with CD4^+^ T cells in inflammatory lesions was further examined. Mice were infected with *M. pulmonis*, and 14 days later, frozen sections of the lung lobes were sectioned and stained with anti-CD11c, anti-F4/80, and/or anti-CD-4 fluorescently labeled Ab.

**Figure 3:**
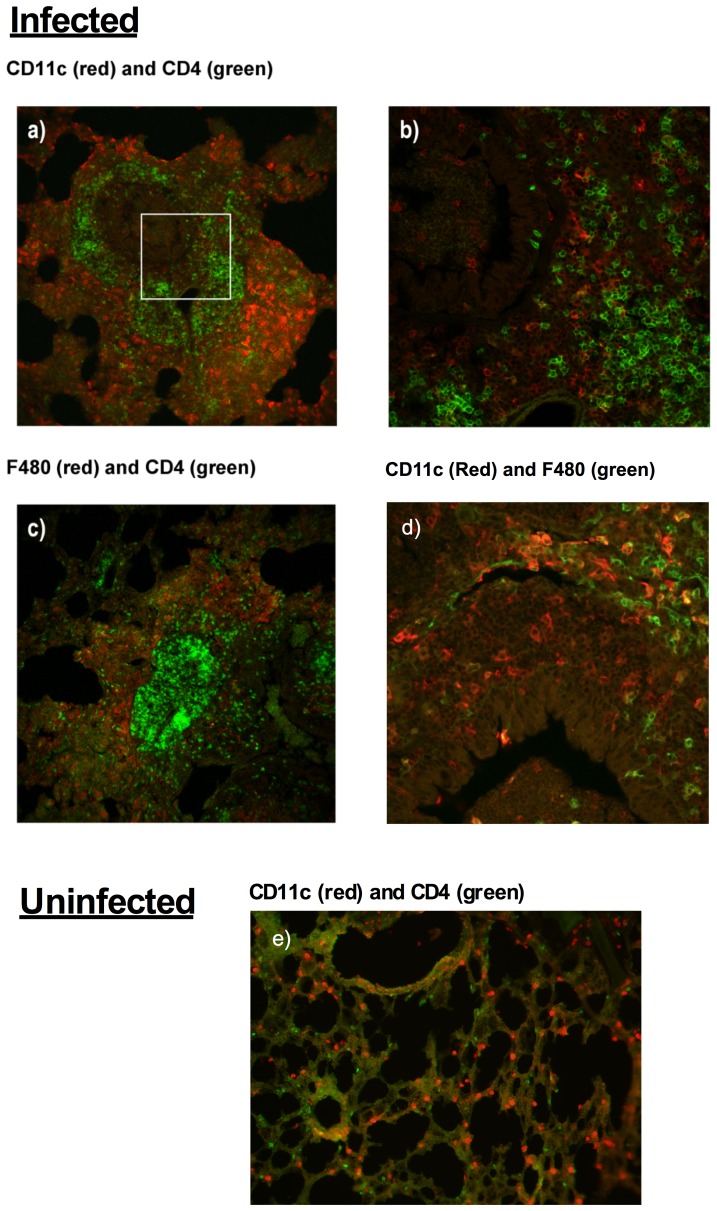
CD11c^+^ cells co-localize with clusters of CD4^+^ T cells in lungs of mice with mycoplasma disease. At 14 days after infection of mice, lungs were collected and frozen sections made. The lungs from infected mice were stained for CD11c, F4/80 and/or CD4 using fluorescently labeled antibodies. (A) Lungs from infected mice were stained for CD11c^+^ (red) and CD4^+^ (green) cells, showing a large number of CD11c^+^ cells surrounding a CD4^+^ T cell region of an inflammatory infiltrate. The white square in image (A) indicates the area from which image (B) was taken. (B) CD11c^+^ cells were also co-localized with CD4^+^ cells within the central region of the inflammatory infiltrates. Lungs from infected mice were also stained for (C) F4/80^+^ (red) and CD4^+^ (green) cells to show that macrophages were also located within the inflammatory lesions, surrounding a central CD4^+^ T cell region. (D) F4/80^+^ (green) and CD11c^+^ (red) cells in the lungs of infected mice were both found in peripheral region of the inflammatory lesions, but CD11c^+^ cells were more frequently also found in the central area. E) The lungs from uninfected mice were stained with CD11c^+^ (red) and CD4^+^ (green), as controls. Unlike infected lungs, the cells were evenly distributed with little, if any, accumulations of inflammatory cells present. Lung sections stained with isotype control antibodies did not shown reaction (not shown). These images were obtained using confocal microscopy, and brightness and contrast adjusted similarly to show the red and green staining in each of the images. These were representative images collected from two experiments and a total of 6 mice.

As shown in Fig. 3, both CD11c^+^ DC and F4/80^+^ macrophages were localized within inflammatory infiltrates within the lungs of infected mice. In the center of these infiltrates large numbers of CD4^+^ T cells were found. Both APC populations were found on the periphery of the infiltrate, surrounding the accumulated CD4^+^ T cells. CD11c^+^ cells were found in the center of the infiltrates, and were co-localized and in contact with CD4^+^ T cells. There were few double positive cells localized in these areas; however, alveolar macrophages appeared to express CD11c, as previously reported [38]. Lung sections stained with isotype control Ab did not show any reaction (Data not shown). In addition, each of the APC populations was evenly dispersed within lungs sections from uninfected mice, as well as lacked areas of Th cell clusters. Thus, both CD11c^+^ DC and F4/80^+^ macrophages contributed significantly to the cellular make up of the inflammatory infiltrates characteristic of mycoplasma disease. Although their distributions were similar, there were indications that DC (CD11c^+^ cells) more frequently were in contact with accumulating CD4^+^ Th cells in these lesions.

CD8^+^ T cells are able to dampen the severity of the inflammatory lesions associated with mycoplasma disease [14], and dendritic cells and other antigen presenting cells can also stimulate CD8^+^ T cells. However, immunofluorescent staining of lungs from mycoplasma infected mice showed that CD8^+^ T cell were few in number and did not localize to the areas of inflammatory infiltrates. In preliminary studies, there was little evidence to suggest that interactions between CD8^+^ T cells and either CD11c^+^ or F4/80^+^ cells occurred frequently within lung tissue (Data not shown).

### Pulmonary CD11c^+^ F4/80^−^ and CD11c^−^ F4/80^+^ cells differ in their cytokine mRNA profiles before and after mycoplasma infection

CD11c^+^ DC and F4/80^+^ macrophage populations were localized in the airway associated mononuclear cell infiltrates, which is a characteristic inflammatory lesion of mycoplasma disease [2], [5]–[8]. To further examine the potential activity of these two cell populations, the expression of different cytokine, chemokine and receptor mRNAs were determined in CD11c^+^ F4/80^−^ and CD11c^−^ F4/80^+^ cells isolated from the lungs of mice 14 days after *M*. *pulmonis* infection. As controls, cells were collected from lungs of mice inoculated with mycoplasma broth. Total RNA was isolated from each cell population and used to probe membrane based cDNA cytokine and chemokine microarrays. The relative levels of cytokine mRNA expression were compared between pulmonary CD11c^+^ F4/80^−^ and CD11c^−^ F4/80^+^ cells from control mice, as well as within each cell population from control and infected mice.

As shown in Table 2, CD11c^+^ F4/80^−^ and CD11c^−^ F4/80^+^ cells from lungs of control (broth-inoculated) mice have different profiles of cytokine, chemokine, and receptors mRNAs. There were 7 genes higher in cells macrophage-enriched (CD11c^−^ F4/80^+^) cells as compared to DC-enriched (CD11c^+^ F4/80^−^) cells. In CD11c^+^ DC cells, there were 10 genes higher than in F4/80^+^ macrophages. Thus, CD11c^+^ DC and F4/80^+^ macrophages from uninfected mice had different cytokine/chemokine mRNA profiles.

**Table 2:** Comparisons in mRNA levels of cytokines and receptors in pulmonary CD11c^+^ F4/80^−^ and CD11c^−^ F4/80^+^ cells in response to *M. pulmonis* infection.^ab^

Genes	Comparison of mRNA levels in control mice		mRNA changes in CD11c^+^ F4/80^−^ cells after *M. pulmonis* infection		mRNA changes in CD11c^−^ F4/80^+^ cells after *M. pulmonis* infection	
	F4/80^+^ cells c	CD11c^+^ cells	Increased^d^	Decreased	Increased	Decreased
CCL2	2.9					
CCL3					2.5	
CCL4	2.2		3.0			
CCL8					6.1	
CCL17		11.6		3.0		
CCR1			2.2			
CCR2	3.9					3.9
CCR7				3.1		
Cx3cr1	2.6					2.7
CxCL4	2.3					
CxCL11			3.5			
CxCL15						
FcεR1g	2.1		2.3			
FcγR1					2.3	
IL-1R1		2.3				
IL-11						
IL-1Rn			2.8		3.9	
IL-1α					3.0	
IL-2Rß		4.3				
IL-2Rγ				2.3		
IL-6		4.9		2.6	3.5	
IL-6ra		3.4		2.4	3.5	
IL-6st				2.6		
IL-10	4.0		2.8			
IL-12a						2.5
IL-12b		7.2		5.0		
IL-15					2.2	
IL-18		2.4				2.2
Ltb		3.8			4.9	
MIF					2.5	
Nos2			6.1		12.6	
Scye1						
Spp1		2.2	4.9		3.2	
TLR2					2.3	
TNF					2.1	
Xcl1		3.4				

a Microarrays were used to compare mRNA expression between lung CD11c^+^ F4/80^−^ and CD11c^−^ F4/80^+^ cells from control (broth-inoculated) or *M. pulmonis* infected mice.

b There no significant differences found in the expression of the following mRNA: Blr1, C3, CCL6, CCL7, CCL19, CCL21a, CCR9, CxCL1, CXCL10, CxCL13, CxCL14, CxCL15, IFN-α 2, IFN-γ, IL-11, IL-8ra, IL-10Rb, IL-16, Itgam, Itgb2, Scye1, TGF-ß1, and Tollip.

c Fold greater of mRNA signal intensity as compared to other cell population. Data represent average value of two individual experiments.

d Fold increase or decrease in cytokine mRNA levels in cells isolated from infected mice from the same cell type isolated from control mice.

After infection, there were changes in the patterns of cytokines and chemokine mRNA expressed by pulmonary DC and macrophages. Using the arrays, there were both increases and decreases in cytokine/chemokine mRNA levels in each of these cell populations. The levels of 7 cytokine mRNAs were reduced, e.g. IL-12b (Table 2), but not eliminated, in CD11c^+^ cells recovered from *M. pulmonis* infected mice, as compared to control (broth-inoculated) mice, whereas there were 8 cytokine mRNAs that were increased, e.g. IL-10, CCL4, and CXCL11. There was a different pattern of cytokine mRNA altered in macrophages recovered from the lungs of infected mice. Four cytokine mRNAs were reduced, e.g. IL-12a and IL-18, but not eliminated, as compared to F4/80^+^ macrophages from control mice. There were 14 cytokine mRNAs that were increased in these cells. These included a number of proinflammatory cytokines, e.g. CCL8, IL-6, IL-1α, Ltb, MIF, and TNF. There were only 3 mRNAs (e.g. IL-1Rn, Nos2 and Spp1) in common between the two cell populations that increased expression, and their function is also typically associated with inflammatory responses. Thus, the patterns of cytokine and chemokine mRNAs produced were consistent with the inflammation, including the recruitment and activation of T cells, but the patterns were also unique to each cell population prior to as well as in response to infection, suggesting potentially different functional activities.

### CD11c^+^ F4/80^−^ DC are the most potent antigen-presenting cells in lungs of mycoplasma-infected mice

To examine their capacity to foster mycoplasma-specific T cell responses, CD11c^−^ F4/80^+^macrophages, CD11c^+^ F4/80^−^ DC and F4/80^−^ CD11c^−^ double negative cells were isolated from lungs of infected and control mice to be used as potential APC *in vitro*. In addition, T cells were purified from the lungs of infected mice and used as the source of mycoplasma-specific T cells, while T cells from broth-inoculated mice were control T cells. The T cells were co-cultured with the APC in the presence and absence of mycoplasma antigen (Ag). IFN-γ levels in culture supernatants were used to monitor T cell responses at 3 days after stimulation.

**Figure 4:**
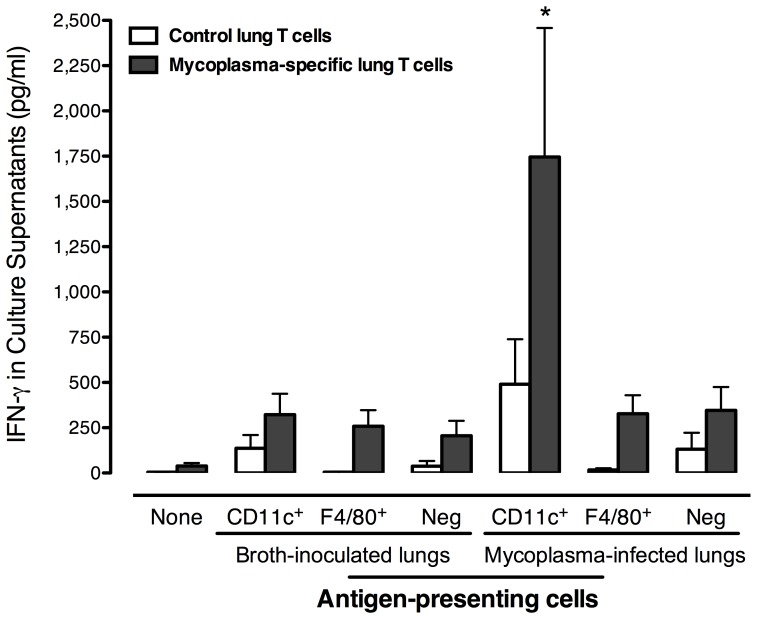
CD11c^+^ F4/80 ^−^ **cells are the most potent antigen-presenting cells in lungs of mycoplasma-infected mice.** CD11c^−^ F4/80^+^ (F4/80+), CD11c^+^ F4/80^−^ (CD11c+), and CD11c^−^ F4/80^−^ (Neg) cells were isolated from lungs of infected and control (broth-inoculated) mice to be used as potential antigen presenting cells. The pulmonary T cells from infected mice (mycoplasma-specific T cells) or from broth-inoculated mice (control T cells) were co-cultured with the APCs in the presence of mycoplasma antigen, and 3 days later, IFN-γ levels in culture supernatants were measured. Cells from 15 naïve mice or 5 infected mice were pooled to obtain sufficient cell numbers for each experiment. Bars represent the mean ± SEM of four independent experiments with each experimental condition done in duplicate in each experiment. * indicates a significant difference (*P*≤0.05) in IFN-γ responses between broth control and infection groups.

As shown in Fig. 4, IFN-γ responses were similar in T cell cultures supplemented with APCs from control mice and stimulated with mycoplasma Ag, regardless of the APC population. However, T cell cultures containing CD11c^+^ F4/80^−^ DC from lungs of infected mice produced significantly higher levels of IFN-γ than any other potential APC population. Antigen-stimulated cultures containing T cells alone did not have significant responses, as well as APC alone (data not shown). No responses were found in the absence of Ag (data not shown).

**Figure 5:**
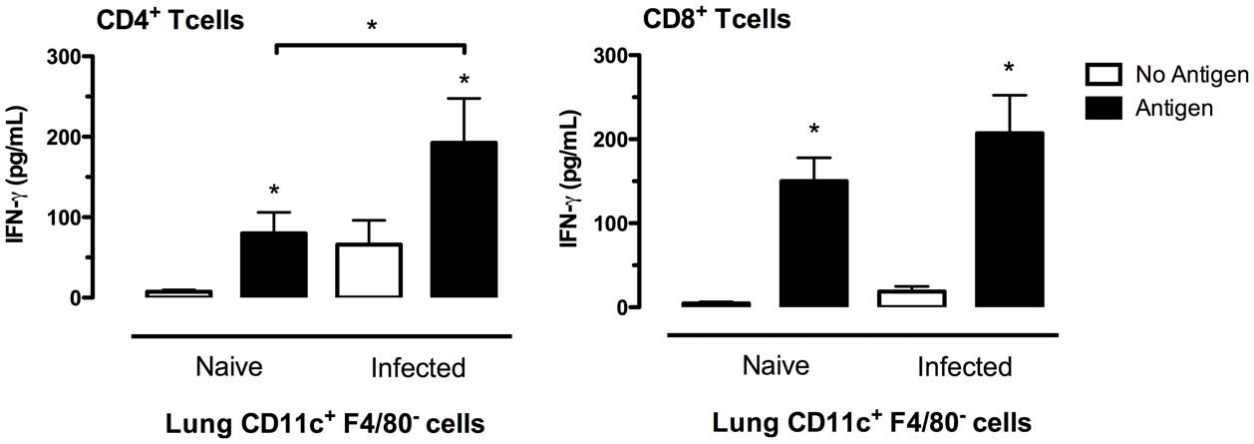
CD11c^+^ F4/80 ^−^ **cells are capable of support both CD8^+^ T cell and CD4^+^ T cell responses against mycoplasma.** CD11c^+^ F4/80^−^ cells were isolated from lungs of infected and control (broth-inoculated) BALB/c mice to be used as antigen presenting cells. Mycoplasma-specific CD8^+^ T cells and CD4^+^ T cells were isolated from the lungs of infected BALB/c mice, and were co-cultured with the CD11c^+^ F4/80^−^ cells in the presence and absence of mycoplasma antigen. Four 4 days later, IFN-γ levels in culture supernatants were measured. Cells from 15 naïve mice or 5 infected mice were pooled to obtain sufficient cell numbers for each experiment. Bars represent the mean ± SEM of five independent experiments with each experimental condition done in at least duplicate in each experiment. * without a bar indicates a significant difference (*P*≤0.05) in IFN-γ levels between cultures stimulated with and without Ag. A bar with an * indicates difference between responses in the presence or absence of antigen. The lines with * indicates difference between the two indicated groups.

To determine whether pulmonary DCs have the capacity to promote activation of mycoplasma-specific CD8^+^ T cells and Th cells, we repeated the above studies using purified T cell populations isolated from the lungs of mycoplasma-infected BALB/c mice. As shown in Fig. 5, DC supported both CD8^+^ T cell and CD4^+^ T cell responses to Ag. However, the response of CD4^+^ T cells was significantly greater when co-cultured with DC from infected mice. No responses were found in the absence of Ag.

## Discussion

Mycoplasma respiratory disease is immunopathologic [46], [47]. It is clear that elements of the adaptive immune response contribute to the pathology, while some responses are protective against *M. pulmonis* infections. Pulmonary T cell activation, and the mechanisms that regulate these responses, is clearly instrumental in the pathogenesis of mycoplasma respiratory disease of the lower respiratory tract [14], [48]. Because of their central role in the activating and regulating T cell responses, APC likely participate in determining whether immune-mediated pathology or protection develops due to mycoplasma respiratory infection, but there is little information on the role of APC populations, particularly DC, during generation of immune and inflammatory responses in mycoplasma respiratory disease.

The numbers of DC and macrophages in the lungs of mice increased during the pathogenesis of mycoplasma respiratory disease. There was about a 3- to 4-fold increase in the numbers of CD11c^+^ DC and F4/80^+^ macrophages in the lungs of mice by 14 days after infection. There was also a significant increase in the numbers of double positive (CD11c^+^ F4/80^+^) cells, which were found to represent alveolar macrophages in another report [14]. The increase in CD11c^+^ cells is consistent with studies demonstrating increasing numbers of DC in lungs in other inflammatory diseases [29]–[31]. The major population of DC that increased expressed CD11b, consistent conventional DC. Additionally, the increases in APC populations due to mycoplasma infection were not apparent at 7 days after infection (data not shown). Our previous studies [14] similarly demonstrate that the numbers of CD4^+^ Th cells and CD8^+^ T cells increase in lung between day 7 and 14 after infection. Thus, there is a concomitant increase in pulmonary DC and macrophage numbers with T cell responses after mycoplasma infection. Furthermore, this time frame coincides with the development of the chronic inflammatory response of the airways in response to the infection. Taken together, there are indeed changes in CD11c^+^ DC and macrophage populations in lungs after mycoplasma infection that accompany development of T cell-mediated inflammation, suggesting a role for these APC in maintaining and modulating T cell responses during the pathogenesis of pulmonary inflammatory disease due to mycoplasma infection.

As the numbers of DC and macrophages increased, there was a change in the populations of DC and macrophages during disease pathogenesis. By 14 days post-infection, expression of MHC II was higher on CD11c^+^ DC, as well as a greater percentage of DC were CD40^+^. Co-stimulatory molecules CD80 and CD86 were also expressed in conjunction of MHC II, especially on CD11^+^ DC cells. There were differences in the expression MHC II, CD40 and CD86 between the naïve APC populations, and changes in expression of these markers suggested that at least a portion of each of the cell populations responded to the infection. In support of activation of APC in lungs in response to infection, there were differences in cytokine mRNA profiles in both CD11c^+^ DC and F4/80^+^ macrophages isolated from lungs of naïve (control) and infected mice. After infection, both DC and macrophage populations also showed an increased expression of cytokine or chemokine mRNA's involved in inflammation (e.g. Nos2, Spp1, TNF, Ltb, MIF, IL-6 and IL-1α), and interestingly they had increased expression of cytokine mRNA's that can function in the recruitment and activation of T cells (e.g. CCL4, CCL8, CxCL11 and IL-15). In fact, previous studies from our lab demonstrated that CCL4 and CCL8 are likely involved in the recruitment of T cells into lesions [36], and the results in the current study suggest that DC and macrophage populations are sources of these chemokines. In any case, it is clear that both macrophages and DC in the lungs are activated or mature as a result of mycoplasma infection, and their differing profiles of cytokine mRNA expression suggest that pulmonary macrophage and DC populations play different roles in modulating immune and inflammatory responses to mycoplasma. In fact, pulmonary CD11c^+^ DC from mycoplasma-infected lungs were more potent antigen presenting cells than those from control lungs in stimulating mycoplasma-specific T (CD4^+^ Th) cell responses *in vitro*. Although other pulmonary cell populations had some activity in supporting T cell responses, DC from diseased lungs were significantly more effective.

Both F4/80^+^ macrophages and CD11c^+^ DC were localized in the inflammatory airway infiltrates typically associated with mycoplasma respiratory disease. Both cell populations surrounded a central Th cell region of the inflammatory infiltrates. The Th cells in the center were frequently co-localized with CD11c^+^ cells. Previous studies demonstrate that a population of Th cells mediates the proinflammatory responses in lungs [14]. The current results demonstrate that CD11c^+^ DC are potent stimulators of mycoplasma-specific Th cell responses *in vitro*, and DC co-localize with Th cells in the pulmonary lesions of mice infected with mycoplasma. In ongoing studies, the priming of mice with mycoplasma Ag-pulsed DC stimulates Th cell responses *in vivo* and results in more intense lymphoid infiltration into the lungs after subsequent infection with mycoplasma (N. Dobbs, X. Zhou, M. Pulse, L.M. Hodge and J.W. Simecka, Unpublished results). Although CD11c^+^ DC were shown to also support mycoplasma-specific CD8^+^ T cell activation, there were relatively few CD8^+^ T cells located within the inflammatory infiltrates and interactions between these cells with either CD11c^+^ DC or macrophages were not consistently found. Thus, DC-Th cell interactions in the lung appear to occur *in vivo*, and these studies suggest that DC facilitates the activation of effector Th cells that contribute to the inflammatory responses associated with mycoplasma respiratory disease.

Overall, the present studies demonstrate that during the pathogenesis of mycoplasma lung disease the populations of DC and macrophages shift from an “inactive” to an “active or mature” state. After infection, DC from the lungs of infected mice became most capable of stimulating mycoplasma-specific T cell responses *in vitro*, and DC co-localized with Th cells in inflammatory infiltrates in the lungs of infected mice. Thus, DC does appear to be major APC population responsible for T cell stimulation in the lungs of mycoplasma-infected mice, and these *in vivo* interactions likely contribute to the immune responses that impact disease pathogenesis. Among these DC, conventional DC are the major contributer to the immune responses. Although macrophages are unlikely to be as effective in directly stimulate T cell responses, macrophages are also associated with the inflammatory lesions and are localized primarily on the periphery of the cellular infiltrates. These results suggest that macrophages do have a significant but different impact from DC on the generation and/or maintenance of host responses associated with mycoplasma disease through the production of cytokines and other factors. Further studies are needed to elucidate the role of each of these cell populations and the cytokines produced in the pathogenesis of mycoplasma disease. Information gained from these studies provides important insight into the immune mechanisms involved in disease pathogenesis and will likely support the development of effective vaccines against mycoplasma respiratory diseases.

## Materials and Methods

### Ethics statement

University of North Texas Health Science Center Institutional Animal Care and Use Committee (IACUC) approved these animal studies.

### Mice

Female mice, tested to be virus- and mycoplasma-free, were used in these studies at 6–12 weeks of age. C3H/HeN mice were used and obtained from Harlan Sprague-Dawley (Indianapolis, IN), except BALB/c mice (Harlan Sprague-Dawley) were used as indicated. BALB/c mice and C3H/HeN mice have similar susceptibilities and cytokine mRNA responses to mycoplasma infection [36], [49]. During the studies, mice were housed in sterile microisolator cages supplied with sterile bedding, food, and water given *ad libitum*. Before experimental infection, mice were anesthetized with an i.m. injection of ketamine/xylazine.

### Mycoplasma

The UAB CT strain of *M. pulmonis* was used in all experiments. Stock cultures were grown, as previously described [50]. Nasal-pulmonary inoculations of 10^5^ CFU/20 µl of mycoplasma were given for experimental infections.

### Pulmonary mononuclear cell isolation

Lungs were perfused with phosphate buffered saline (PBS) without magnesium or calcium to minimize contamination of the final lung cell population with those from blood. The lungs were separated into individual lobes, finely minced and digested at 37°C in RPMI 1640 (HyClone Laboratories, Logan, UT) medium containing 300 U/ml *Clostridium histolyticum* type I collagenase (Worthington Biochemical, Freehold, NJ), 50 U/ml DNase (Sigma-Aldrich, St. Louis, MO), 10% Fetal Bovine Serum (FBS) (HyClone Laboratories), HEPES, and antibiotic/antimycotic solution (Life Technologies, Grand Island, NY). The digestion mixture was passed through a 250-µm nylon mesh to remove undigested tissue. RBCs were lysed with ammonium chloride-potassium carbonate lysis buffer. Mononuclear cells were purified from cell suspensions by density gradient centrifuge on Histopaque-1077 (Sigma-Aldrich, St. Louis, MO) and further used for flow cytometry analysis, as well as cell purification.

### Flow cytometry

1∼2×10^6^ of pulmonary mononuclear cells were suspended in staining buffer (PBS, 1% FCS, 2 mM EDTA). Prior to staining, cells were incubated for 30 min with anti CD16/CD32 Fc Block (clone, 2.4G2, BD PharMingen, San Diego, CA), then stained with pre-optimized concentrations of appropriate antibody (Ab) for 1 hour at 4°C in the dark. The Abs used to characterize sub-populations of dendritic cells/macrophages in mouse lung were: FITC rat anti-mouse MHCII (I-A/I-E; 2G9), PE hamster anti-mouse CD11c (HL3), PerCP-Cy5.5 hamster anti-mouse CD80 (16-10A1), APC rat anti-mouse CD40 (23-Mar), Alexa Fluor 700 rat anti-mouse CD11b (M1/70), PE-Cy7 rat anti-mouse CD86 (GL1), (BD PharMingen), APC-Alexa Fluor 750 rat anti-mouse F4/80 (BM8), PerCP rat anti-mouse B220 (RA-6B2), and APC rat anti-mouse CD8α (53-6.7) (Invitrogen, Carsbad, CA). Data were acquired by BD LSRII Cytometer (BD Biosciences, San Jose, CA) and analyzed by FlowJo software (Tree Star, Ashland, OR). CompBeads Anti-Rat/Hamster Ig, κ particles (BD PharMingen) were stained with each antibody and compensation was calculated using FlowJo. To characterize the APC populations, at least 10^6^ cells were analyzed. The criterion of CD11c and F4/80 positive populations were determined by Fluorescence Minus One (FMO) method [51] plus isotype, in which the anti-CD11c or anti-F4/80 antibody was replaced by relevant isotype control in the seven color staining panel. Gates were set to result in less than 1% background cells for each gate using FMO plus isotype.

### Immunofluorescent staining of pulmonary cell populations

Lungs were inflated through trachea and fixed with 4% paraformaldehyde (Sigma Chemical Co., St Louis, MO, USA) for 2 to 4 hours, submerged in 30% sucrose solution overnight, then embedded in Tissue-Tek OCT compound (Sakura Finetechnical, Torrance, CA, USA). Lungs were sectioned at a 15 µm thickness using an Ultrapro 5000 cryostat (Vibratome, Saint Louis, MO) and mounted on FisherBrand SuperFrost/plus microscope slides (Fisher Scientific, Hampton, NH). Tissues on slides were fixed with cold acetone for 5 min, blocked by 10% fetal bovine serum (FBS) in PBS, co-incubated with biotinylated anti-mouse CD11c (HL3) Ab (BD PharMingen) or biotinylated anti-mouse F4/80 Ab in combination with AlexaFluor 488 conjugated rat-anti-mouse CD4 (Invitrogen, Caltag laboratories, Carlsbad, CA 92008), followed by incubation with anti-biotin AlexaFluor 594 secondary Ab (Invitrogen, Molecular Probe, Carlsbad, CA). The images were observed by AX70 Olympus Fluorescence microscope and captured by DP70 camera (Olympus, Center Valley, PA) or Zeiss LSM 410 Confocal microscope (Carl Zeiss Inc, Thornwood, NY).

### Cell isolation and purification

Cell populations were purified using paramagnetic bead-conjugated Ab and an autoMACS (Miltenyi Biotec, Auburn, CA) following manufacturer's instructions. CD11c^+^ cell selection was done using CD11c (N418) MicroBeads (Miltenyi Biotec). F4/80^+^ cells selection was done in a two-step process. Mononuclear cells were incubated with biotinylated-anti-mouse F4/80 Ab at a 1∶40 dilution (Invitrogen, Caltag laboratories, Carlsbad, CA) followed by anti-biotin MicroBeads (Miltenyi Biotec). In order to get pure CD11c^+^ or F4/80^+^ without CD11c^+^ F4/80^+^ double positive cells, pulmonary mononuclear cells were divided into two parts, one for CD11c^+^ cells isolation and one part for F4/80^+^ cells isolation. For CD11c^+^ F4/80^−^ cell purification, mononuclear cells were first depleted of F4/80^+^ cells using F4/80^+^ cells selection beads and CD11c^+^ cell were subsequently isolated. Similar procedure was applied to obtain F4/80^+^ CD11c^−^ cells.

After removal of CD11c^+^and F4/80^+^ cells, pulmonary T cells were isolated using the Pan T cell Isolation Kit (Miltenyi Biotec). All non-T cells are removed using a cocktail of biotin-conjugated Abs followed incubation with Anti-Biotin MicroBeads.

For the studies using purified CD4^+^ T cells and CD8^+^ T cells, the purified T cells were depleted of residual APC by removing MHC II^+^ cells (Miltenyi Biotec). CD8^+^ T cells were positively selected from the purified T cell population (positive selection kit, Miltenyi Biotec), and the remaining CD8 negative cells contained CD4^+^ T cells.

When depletion of cells was used in cell isolation from lungs, there were <2% residual cells remaining in the depleted cell population. Positive selection of cells resulted in ≥85% purity for CD4^+^, F480^+^ or CD11c^+^ cells and ≥65% for CD8^+^ cells. We were unable to detect CD3^+^ cells in any of the APC populations.

### RNA extraction

RNA from purified CD11c^+^ or F4/80^+^ cells was isolated using Trizol RNA isolation reagent as recommended by the manufacturer's instructions (Invitrogen Life Technologies, Carlsbad, CA) and was quantified by spectrophotometry (GeneQuant II; Amersham Pharmacia Biotech, Piscataway, NJ).

### Microarrays

Similar to our previous studies [15], [36], Oligo GEArray^R^ Mouse Inflammatory Cytokines and Receptors Microarrays (SuperArray Bioscience Corp., Frederick, MD) were used to evaluate the expression of 113 key genes involved in inflammatory responses. Biotinylated-cRNA probes were prepared using AmpoLabeling-AMP™ 2.0 kit (SuperArray). Total RNA from purifed pulmonary CD11c^+^ or F4/80^+^ cells collected from broth inoculated (control) mice or *M. pulmonis* infected mice from three individual sets of experiments. An equal amount RNA from each of experiment was mixed to total 3 µg RNA, and this used as template for probe synthesis. After hybridization of probes, alkaline phosphatase-conjugated streptavidin at a 1∶8,000 dilution was incubated with membrane for 10 min. The signals were developed in CDP-Star chemiluminescent substrate for 3 minutes, and the image was recorded using a CCD camera (FluorChemTM 8900 Imaging System, Alpha Innotech, San Leandro, CA). The image data were analyzed by web based GEArray Analysis Suite supported by SuperArray Inc, and the gene expression results were normalized to β-actin gene expression. Changes in gene expression were considered significant only if there was at least an average of ≥2-fold difference, with no value of ≤1.5 fold in either individual experiment [36].

All data is MIAME compliant and that the raw data has been deposited in a MIAME compliant database (GEO, Series entry number GSE33369).

### Preparation of *M. pulmonis* antigen (Ag)

A crude preparation of *M. pulmonis* membranes was used for *in vitro* mycoplasma-specific T cell stimulation and prepared as previously described [52], [53]. The protein concentration was determined by standardization using a Bradford protein assay (BioRad, Hercules, CA).

### *In vitro* stimulation of Ag-specific T cells

T cell populations (2×10^5^/well) purified from lungs of mycoplasma-infected or broth-inoculated mice were cultured in the presence or absence of APC populations (5×10^4^/well) in Microtest^™^ U-bottom 96-well culture plates (Becton Dickinson Labware, Franklin Lake, NJ) in RPMI 1640 medium (HyClone Laboratories) with 10% FBS and antibiotic/antimycotic (Life Technologies). Lymphoid cells were stimulated at 37°C and 5% CO_2_. Cells were stimulated with or without 5 µg/ml of mycoplasma Ag was presence or absence in the culter to observe antigen specific reaction. Supernatants were collected three days later and stored at −80°C until assayed for IFN-γ levels.

### IFN-γ ELISA

The levels of IFN-γ were measured by capture ELISA using the IFN-γ BD OptEIA™ mouse ELISA kit (BD Biosciences, Bedford, MA) per the manufacturer's instructions. 3,3'5,5'-tetramethylbenzidine substrate (TMB, Moss, Pasadena, MD) was used to reveal the reaction. Plates were read using MX80 plate reader (Dynatech, Chantilly, VA) at an absorbance of 630 nm. Cytokine levels were determined by comparison with standard curves generated from murine recombinant cytokines after log/log quadratic linear regression analysis using Revelation 2.0 software (Dynatech).

### Statistical Analysis

Data was evaluated by ANOVA, followed by Fisher Protected Least Square Differences Multigroup comparison. These analyses were performed using the StatView (SAS Institute, CARY, NC) or Prism (GraphPad Software, Inc., La Jolla, CA) computer program. Cell numbers were logarithmically transformed prior to analyses. A *P* value ≤0.05 was considered statistically significant.
